# Supplementary material for: The Incidence and Severity of Post-Vaccination Reactions after Vaccination against COVID-19
Source: Vaccines (Basel). 2021 May 13;9(5):502. doi: 10.3390/vaccines9050502 (PMC8152224; doi:10.3390/vaccines9050502)
Supplement: Supplementary file 1 [file vaccines-09-00502-s001.zip › vaccines-1182727-supplementary.pdf]

Assessment of vaccination against COVID 19 in selected groups in stage zero of the National Immunization Program

Please complete an anonymous questionnaire consisting of 20 short questions regarding, among others, getting COVID 19, post-vaccination reactions after receiving the first and second doses of the COVID19 vaccine, and motivation to proceed with vaccination. The survey is conducted by the team of the Chair and Department of Pharmacology at the Medical University of Wrocław.

1. Belonging to a group that determines vaccination group 0 of the National Immunization Program, more than one answer can be marked.

☐Academic teacher employed at a medical university

☐Doctor

☐Nurse

☐Pharmacist

☐Pharmaceutical technician

☐PhD student

☐Student of medical university

☐ Other

2. Education:

☐Higher education

☐Secondary education

☐Professional education

☐PhD student

☐Student

3. Sex:

☐woman

☐man

4. Age

☐ 19-30 years

☐ 31-40 years

☐ 41-50 years

☐ 51-60 years

☐ above 60 years

5. Have you suffered from COVID 19 confirmed by a test?

☐Yes

☐No

☐No sure

6. Have you ever had COVID 19 that was not confirmed by the test?

☐Yes

☐No

☐No sure

7. What was your COVID 19 mileage?

☐ I DIDN'T HAVE COVID19

☐ Asymptomatic

☐ MILD - mild upper respiratory tract symptoms, elevated body temperature not exceeding 38 ° C, cough or shortness of breath, treatment at home

☐MODERATE – fever > 38 ° C, persistent dry cough, dyspnoea, imaging lung involvement, outpatient treatment

☐Severe – hospital treatment

☐VERY HEAVY – hospital treatment with the use of a respirator

8. Why have you given your consent to be vaccinated against COVID19? You can mark more than one answer.

☐ I do not consent to any voluntary vaccination

☐ I do not consent to vaccination against COVID 19

☐ Occupation related knowledge about COVID19

☐Concern for my health

☐ Presence of other chronic diseases

☐Caring for the health of loved ones

☐Concern for the health of patients

☐History of severe COVID 19 disease

☐Encouragement from the employer

☐High risk of infection due to the current state of the COVID 19 pandemic

☐The ability to travel

☐Encouragement from scientific and medical authorities

☐Media information, including publicity advertisements

9. Did you experience the symptoms listed below after the first dose of the vaccine?

☐No symptoms

☐Severe reaction at the injection site (swelling, redness and soreness)

☐Injection site abscess

☐Soreness and enlargement of the regional lymph nodes

☐Increased body temperature (between 36.6-38 ° C)

☐Fever (over 38 ° C)

☐ Seizures

☐Allergic reaction (hives, tearing and runny nose, generalized rash, erythema multiforme, asthma attack)

☐Anaphylactic shock

☐Diarrhea

☐Blue limbs

☐Nausea

☐Vomiting

☐Cutaneous petechiae

☐Fatigue

☐Bad mood

☐Cough

☐Headache

☐Migraine

☐Swoon

☐Other

10. Did you develop any of the symptoms listed below after receiving the second dose of the vaccine?

☒No symptoms

☐Severe reaction at the injection site (swelling, redness and soreness)

☐Injection site abscess

☐Soreness and enlargement of the regional lymph nodes

☐Increased body temperature (between 36.6-38 ° C)

☐Fever (over 38 ° C)

☐ Seizures

☐Allergic reaction (hives, tearing and runny nose, generalized rash, erythema multiforme, asthma attack)

☐Anaphylactic shock

☐Diarrhea

☐Blue limbs

☐Nausea

☐Vomiting

☐Cutaneous petechiae

☐Fatigue

☐Bad mood

☐Cough

☐Headache

☐Migraine

☐Swoon

☐Other

11. How will you compare the severity of post-vaccination symptoms after taking the first and second doses?

☐ No difference

☐Weaker after taking the second dose

☐Stronger after taking the second dose

12. Which of the following symptoms were more severe after the second dose than after the first dose of the vaccine?

☒No symptoms

☐Severe reaction at the injection site (swelling, redness and soreness)

☐Injection site abscess

☐Soreness and enlargement of the regional lymph nodes

☐Increased body temperature (between 36.6-38 ° C)

☐Fever (over 38 ° C)

☐ Seizures

☐Allergic reaction (hives, tearing and runny nose, generalized rash, erythema multiforme, asthma attack)

☐Anaphylactic shock

☐Diarrhea

☐Blue limbs

☐Nausea

☐Vomiting

☐Cutaneous petechiae

☐Fatigue

☐Bad mood

☐Cough

☐Headache

☐Migraine

☐Swoon

☐Other

13. How do you rate the "strength" of your motivation to vaccinate?

☐Definitely yes

☐Rather yes

☐Rather no

☐Definitely no

14. Are you satisfied with the decision to vaccinate?

☐Yes

☐No

15. How do you assess the impact of the media on the decision to vaccinate against COVID19?

☐Definitely yes

☐Rather yes

☐Rather no

☐Definitely no

16. Will you get vaccinated again against COVID19?

☐Yes

☐No

☐No sure

17. Did you report an adverse vaccination reaction (nop) to the Sanitary Inspection?

☐Yes

☐No

18. What company were you vaccinated with the preparation?

☐Pfizer

☐AstraZeneca

☐Moderna

19. What company would you choose if you could choose a COVID19 vaccine?

☐Pfizer

☐AstraZeneca

☐Moderna

☐Other

20. What technology is the vaccine production based on?

☐No sure

☐The mRNA vaccine on the S protein

☐Vector vaccine on the S protein of adenovirus
